# Early and Timely Surgery for an Atypical Form of Partial Anomalous Pulmonary Venous Return Enabled by a Rare Detection by Detailed Fetal Ultrasound

**DOI:** 10.7759/cureus.77798

**Published:** 2025-01-21

**Authors:** Hiroyuki Nagao, Shintaro Nemoto

**Affiliations:** 1 Department of Pediatrics, Takatsuki General Hospital, Takatsuki, JPN; 2 Department of Pediatric Thoracic and Cardiovascular Surgery, Osaka Medical and Pharmaceutical University Hospital, Takatsuki, JPN

**Keywords:** congenital cardiac surgery, fetal echocardiography, partial anomalous pulmonary venous return, prenatal, sinus venosus asd

## Abstract

While recent advances in fetal echocardiography diagnosis have facilitated the treatment of congenital heart disease (CHD) early in life, many diseases remain overlooked by current recommended ultrasound protocols. Among them, partial pulmonary venous return (PAPVR) is a rare disease for which surgery is indicated; however, it progresses from birth without obvious symptoms. We report a case where we carefully examined the right, left, upper, and lower pulmonary veins (PVs) in more detail than is recommended by standard fetal echocardiography protocols, leading to the early detection of an atypical form of PAPVR. Based on close follow-up after the diagnosis, surgical repair was successfully performed during infancy, resulting in improved right heart enlargement and steady weight gain postoperatively. PAPVR is often overlooked, and its undiagnosed progression can lead to impaired cardiac function in adulthood. As routine fetal ultrasound (FU) examinations frequently omit detailed assessments of PVs, this report highlights the importance of incorporating such detailed evaluations into fetal echocardiography protocols.

## Introduction

Partial anomalous pulmonary venous return (PAPVR) and total anomalous pulmonary venous return (TAPVR) are congenital heart anomalies that involve abnormal connections of the pulmonary veins (PVs). In TAPVR, all PVs connect to the right heart system instead of the left atrium (LA), whereas in PAPVR, only some PVs - typically on the right - abnormally connect to the right heart system. One of the primary purposes of routine fetal ultrasound (FU) is to detect severe congenital heart diseases (CHD) that require prompt treatment immediately after birth, such as TAPVR. To detect TAPVR, visualization of at least one of the left PVs connecting to the LA is a diagnostic criterion in globally recommended FU screenings [1,2].

In PAPVR, however, the right PVs, not on the left as in TAPVR, are the only ones abnormally connecting to the right heart system, and hence the condition is often overlooked by conventional recommendations and diagnosed only after symptoms appear as the disease progresses. Delayed diagnosis can result in the development of significant pulmonary hypertension (PH) and right heart failure. Recognizing and diagnosing PAPVR at an earlier stage allows for timely surgical intervention, preventing complications and improving long-term outcomes. This report highlights the importance of implementing detailed FU assessments extending beyond current recommended protocols to enable earlier diagnosis and timely surgical correction of rare congenital anomalies such as PAPVR.

## Case presentation

**Figure 1:**
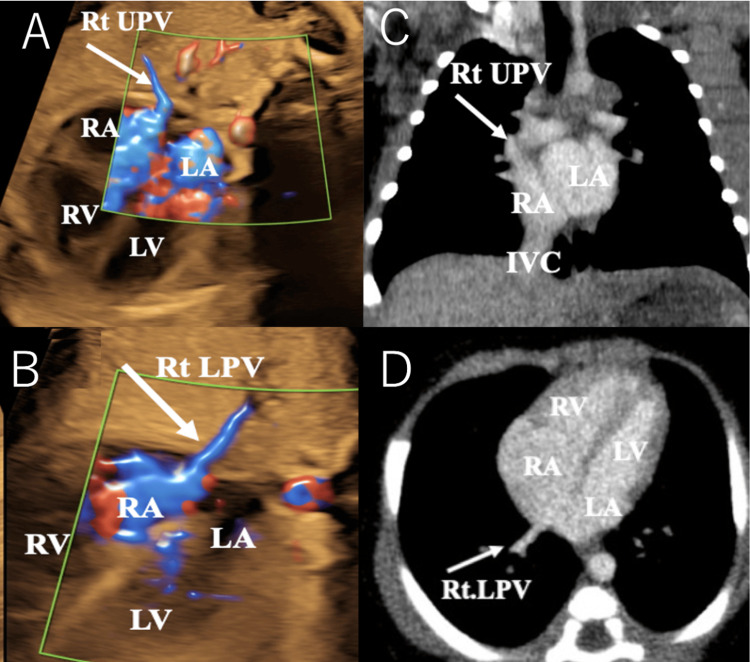
Fetal echocardiography and postnatal contrast-enhanced CT scan images A: Fetal echocardiography showing Rt UPV. B: Fetal echocardiography showing the Rt LPV. C: Postnatal contrast-enhanced CT confirmed that the Rt UPV, which normally drains into the LA, was abnormally connected to the RA. D: Postnatal contrast-enhanced CT confirmed that the Rt LPV, which normally drains into the LA, was abnormally connected to the RA CT: computed tomography; IVC: inferior vena cava; LA: left atrium; LV: left ventricle; RA: right atrium; Rt LPV: right lower pulmonary vein; Rt UPV: right upper pulmonary vein; RV: right ventricle

A pregnant female with a history of epileptic seizures was referred to our hospital for the management of a high-risk pregnancy. Fetal growth was within normal limits. Standard FU follow-up at 35 weeks of gestation revealed PAPVR, where the right PVs were found to connect to the right atrium (RA). The anomalous connection was identified through a detailed evaluation of the PVs using advanced imaging techniques. However, fetal echocardiography could not definitively differentiate between a simple patent foramen ovale and a sinus venosus atrial septal defect (ASD). Based on the findings, we concluded that this case lacked the sinus venosus ASD, characteristic of PAPVR (Figures 1A, 1B).

As the cardiac lesion was not complex and did not require immediate intensive treatment after birth, the mother delivered a female baby (birth weight: 3,295 g) via normal vaginal delivery at 38 weeks and five days of gestation. Postnatal echocardiography confirmed the FU diagnosis but also identified a sinus venosus ASD, which was small and inferior rather than the typical superior location. Contrast-enhanced CT further confirmed the anatomical configurations of all PVs as consistent with the FU findings, with no additional structural cardiac abnormalities detected (Figures 1C, 1D). The left PVs were confirmed to connect to the left atrium, and flow from the left atrium to the left ventricle was visualized, ruling out TAPVR.

**Figure 2:**
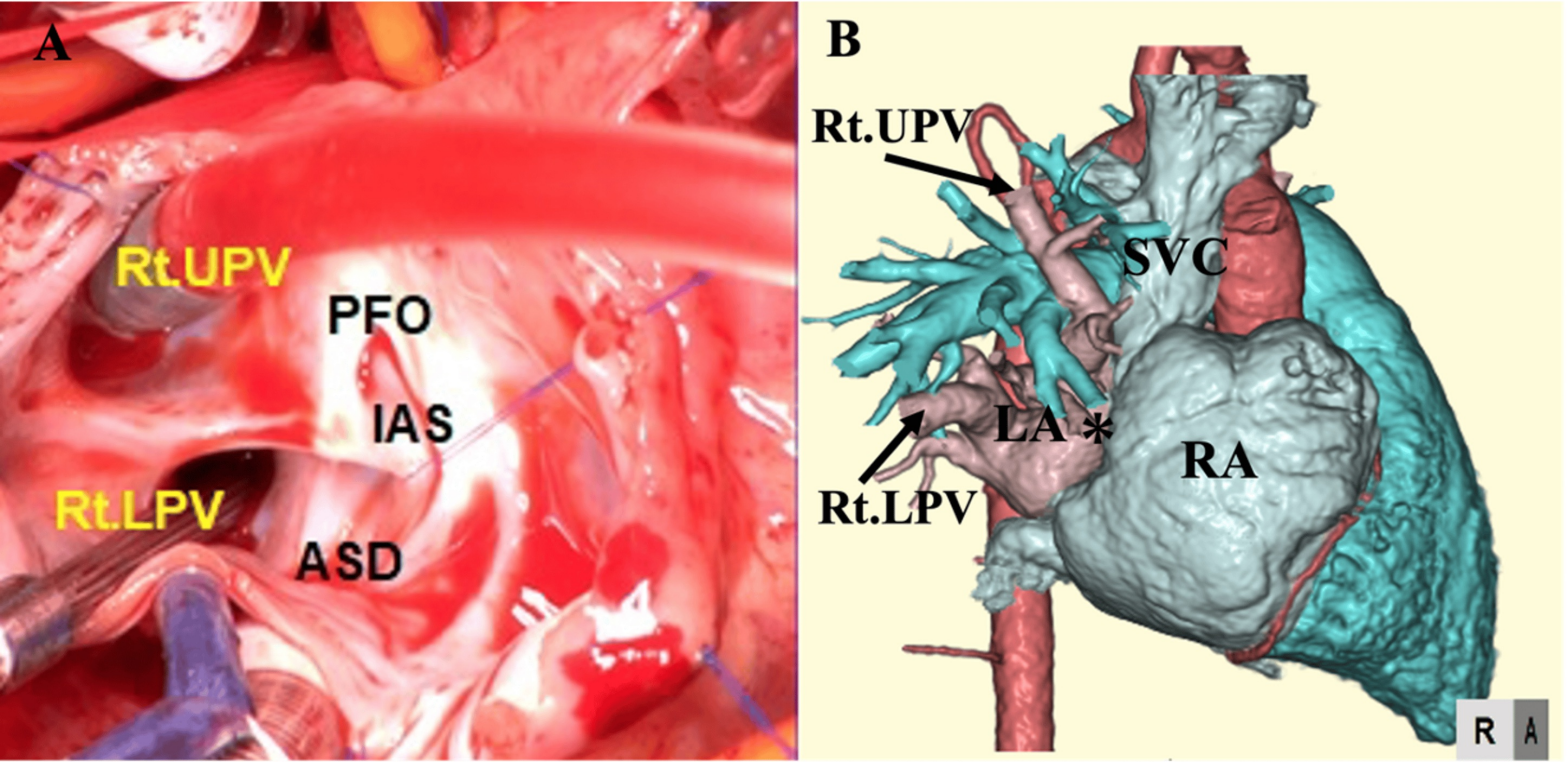
Intraoperative findings and postoperative contrast-enhanced CT images A: Intraoperative findings. B: Postoperative contrast-enhanced CT The flaps indicated by the asterisk (*) represent surgically created structures designed to redirect the anomalous drainage of both the Rt UPV and Rt LPV from the RA to the LA. These flaps are critical in restoring the normal connection of the pulmonary veins to the LA, which ensures that oxygenated blood is delivered to the systemic circulation as intended, rather than mixing with deoxygenated blood in the RA ASD: atrial septal defect; CT: computed tomography; IAS: interatrial septum; LA: left atrium; PFO: patent foramen ovale; RA: right atrium; Rt LPV: right lower pulmonary vein; Rt UPV: right upper pulmonary vein; SVC: superior vena cava

Despite the absence of significant symptoms, serial follow-up echocardiography after discharge revealed progressive enlargement of the right heart system. At seven months of age, the patient underwent cardiac catheterization due to inadequate weight gain, which was attributed to signs of right heart failure. Cardiac catheterization revealed a Qp/Qs ratio of 3.0 and a right ventricular end-diastolic volume that was 181% of normal, indicating significant enlargement of the right heart due to increased pulmonary blood flow. The mean pulmonary artery pressure was 20 mmHg, indicating mild pulmonary hypertension. The intracardiac repair was performed at 10 months of age. During surgery, all right PVs were confirmed to drain into the RA, along with a small inferior sinus venosus ASD and a patent foramen ovale (Figure 2A).

Given the patient’s somatic growth and the need to accommodate future cardiac development, a repair technique using only autologous tissue, specifically septal and right atrial flaps, was chosen (Figure 2B). This approach differed from other methods by avoiding synthetic materials, which may carry additional risks in young patients, and provided an optimal balance between immediate correction and future growth.

Following surgery, the right heart system enlargement resolved, and the patient’s weight gain significantly improved. Preoperatively, the patient’s weight gain was at the 10th percentile; however, postoperatively, it increased to the 50th percentile, reflecting a marked improvement in overall growth and development. Long-term follow-up is necessary to monitor for potential complications, such as stenosis at the rerouted PV sites or recurrence of right heart failure symptoms.

## Discussion

PAPVR is challenging to detect in FU due to its low incidence, accounting for only 0.2-0.7% of CHD cases [3], and the lack of a specific protocol for PAPVR detection, as current recommendations focus on TAPVR [1,2,4]. TAPVR detection protocols prioritize confirming at least one PV draining into the LA, often overlooking the right PVs that are abnormal in PAPVR. This limitation, combined with the rarity of PAPVR and the lack of immediate surgical urgency after birth, results in lower detection rates universally.

Although emergency surgery is not required for PAPVR immediately after birth, untreated cases may develop arrhythmias or heart failure due to pulmonary overcirculation, which could persist into adulthood [5]. Pulmonary overcirculation frequently leads to PH and right-side heart failure. For example, the annual incidence of idiopathic PH ranges from 7.1 to 7.6 cases per million (0.0007%), with a prevalence of 52 cases per million (0.0052%) [6]. However, a cohort study on PAPVR reported a much higher prevalence of PH (9%) confirmed by cardiac catheterization [7]. This reinforces the notion that prolonged untreated PAPVR contributes significantly to PH development.

In this case, cardiac catheterization revealed pulmonary overcirculation with elevated mean pulmonary artery pressure and right ventricular enlargement, meeting the criteria for early surgical intervention. While arrhythmias or heart failure are rare in infancy, accumulated pulmonary overcirculation from PAPVR can predispose patients to these complications later in life. Hence, early diagnosis and timely surgical intervention are necessary regardless of the presence or absence of symptoms. Regarding ideal surgical correction during infancy, it is necessary to take growth potential into account and choose a different technique from those performed in adults [8]. The RA flap technique was chosen to accommodate somatic growth, avoiding synthetic materials that may carry risks in young patients. This approach differs from adult surgical techniques and provides an optimal balance between immediate correction and future growth. Following surgery, right heart enlargement resolved, and the patient’s weight gain significantly improved.

Long-term follow-up is necessary to monitor potential complications, such as stenosis at the rerouted PV sites or recurrence of right heart failure symptoms. Early diagnosis and intervention have been shown to result in low mortality and excellent prognosis in PAPVR cases operated on during infancy [9].

## Conclusions

We described the detection of a rare congenital defect using a more comprehensive FU protocol than currently recommended, enabling early diagnosis and timely surgical correction of an atypical form of PAPVR, a serious condition that often remains undiagnosed until adulthood and can lead to significant PH and right heart failure. This early intervention led to the resolution of right heart enlargement and a remarkable improvement in the patient’s weight gain, increasing from the 10th percentile preoperatively to the 50th percentile postoperatively. Detecting PAPVR using current standard FU guidelines poses significant challenges, and the successful diagnosis of PAPVR through postnatal evaluation is particularly difficult. This report highlights how detailed prenatal screening can help overcome such challenges by enabling early identification of abnormalities and facilitating timely intervention. The report further emphasizes the importance of FU in diagnosing serious CHD and non-urgent conditions requiring surgical correction.
